# Has the free maternal health policy eliminated out of pocket payments for maternal health services? Views of women, health providers and insurance managers in Northern Ghana

**DOI:** 10.1371/journal.pone.0184830

**Published:** 2018-02-01

**Authors:** Philip Ayizem Dalinjong, Alex Y. Wang, Caroline S. E. Homer

**Affiliations:** Faculty of Health, University of Technology Sydney, Sydney, New South Wales, Australia; University of North Carolina at Chapel Hill, UNITED STATES

## Abstract

**Introduction:**

The free maternal health policy was implemented in Ghana in 2008 under the National Health Insurance Scheme (NHIS). The policy sought to eliminate out of pocket (OOP) payments and enhance the utilisation of maternal health services. It is unclear whether the policy had altered OOP payments for services. The study explored views on costs and actual OOP payments during pregnancy. The source of funding for payments was also explored.

**Methods:**

A convergent parallel mixed methods design, involving quantitative and qualitative data collection approaches. The study was set in the Kassena-Nankana municipality, a rural area in Ghana. Women (n = 406) who utilised services during pregnancy were surveyed. Also, 10 focus groups discussions (FGDs) were held with women who used services during pregnancy as well as 28 in-depth interviews (IDIs) with midwives/nurses (n = 25) and insurance managers/directors (n = 3). The survey was analysed using descriptive statistics, focussing on costs from the women’s perspective. Qualitative data were audio recorded, transcribed and translated verbatim into English where necessary. The transcripts were read and coded into themes and sub-themes.

**Results:**

The NHIS did not cover all expenses in relation to maternal health services. The overall mean for OOP cost during pregnancy was GH¢17.50 (US$8.60). Both FGDs and IDIs showed that women especially paid for drugs and ultrasound scan services. Sixty-five percent of the women used savings, whilst twenty-two percent sold assets to meet the OOP cost. Some women were unable to afford payments due to poverty and had to forgo treatment. Participants called for payments to be eliminated and for the NHIS to absorb the cost of emergency referrals. All participants admitted the benefits of the policy.

**Conclusion:**

Women needed to make payments despite the policy. Measures should be put in place to eliminate payments to enable all women to receive services and promote universal health coverage.

## Introduction

Ghana is a West African country with a population of about 24 million [1]. In 2012/13, the country had a mean annual gross household income of GH¢16,645 (US$ 8,446), with a mean per capita income of GH¢5,347 (US$2,713), translating to GH¢14.65 (US$ 7.4) per person per day [2]. Of the ten regions of Ghana, the Upper East region had the lowest mean annual gross income of GH¢7,240.5 (US$3,673.8) and a mean annual per capita income of GH¢1,801.9 (US$914), approximated to be Gh¢4.6 (US$ 2.5) per person per day [2]. Agriculture, forestry and fishing employs about 65% of Ghanaians (dominated by rural population) aged 15 years and above [2]. The average health facility cost for one outpatient visit in Ghana was estimated to be GH¢14 (US$4) at a maternity clinic and GH¢33 (US$10) at a regional referral hospital in 2011 [3]. Overall, Ghana’s per capita expenditure on health was US$32.8 in 2014 [4].

Maternal mortality is a significant concern for Ghana. In 2015, the maternal mortality ratio was 319 per 100,000 live births [5]. Haemorrhage, abortion, hypertensive disorders, infections and ectopic gestation are recorded as the main causes of maternal deaths in the country [6–8]. However, effective and timely antenatal care (ANC) has the capacity to contribute to saving lives of pregnant women and their babies. ANC offers the opportunity for provision of health services, including health education, testing and diagnosis of health problems for treatment and rendering of essential interventions to prevent diseases among pregnant women and their unborn babies [9]. In addition, ANC provides opportunities for communication and support for women, their families and communities during pregnancy and childbirth [9].

Given the benefits, the World Health Organisation (WHO) had outlined some recommendations to be carried out during ANC (Table 1).

**Table 1:** WHO recommendation for improving health of pregnant women [10].

Essential	Situational
• Confirmation of pregnancy • Monitoring of progress of pregnancy and assessment of maternal and fetal well-being • Detection of problems complicating pregnancy (for example, anaemia, hypertensive disorders, bleeding, malpresentations, multiple pregnancy • Respond to other reported complaints • Tetanus immunization, anaemia prevention and control (iron and folic acid supplementation) • Information and counselling on self-care at home, nutrition, safer sex, breastfeeding, family planning, healthy lifestyle • Birth planning, advice on danger signs and emergency preparedness • Recording and reporting • Syphilis testing	• HIV testing and counselling • Antimalarial Intermittent preventive treatment (IPT) and promotion of insecticide treated nets (ITN) • Deworming • Assessment of female genital mutilation (FGM)

The recently released WHO guidelines now recommend a minimum of eight visits for ANC by pregnant women, instead of the four previously recommended. This is to reduce prenatal deaths and generate a positive experience for pregnant women [9]. The new policy however, is likely to have significant resources and human resource challenges in many low to middle income countries and potentially may contribute to further costs for families [11]. An understanding of the implication of the previous recommendation and policy therefore is important as the new recommendation for eight ANC visits are considered and implemented.

In Ghana, there has been a steady decline in ANC visits (4+) from 98.6% in 2011, to 92.2% in 2012, to 90.8% in 2013 and down to 86.7% in 2014 [12]. The downward trend had been attributed to lack of funds for outreach programmes in the communities as well as poor data capture [12]. Some studies in Ghana show that women who are uneducated, poor and live in rural areas tend to access fewer ANC visits compared to their counterparts who are educated, wealthy and are urban dwellers [13, 14]. This again shows the need to better understand the cost implications for families utilising ANC.

### Implementation of the National Health Insurance Scheme (NHIS)

Eliminating out of pocket (OOP) payments is considered one of the best options for promoting the utilisation of health services, including ANC and skilled attendance at childbirth [15–18]. The removal of OOP payments also assist to reduce impoverishment among households [18, 19]. To address the burden of OOP payments and enhance utilisation, in 2003 the Government of Ghana established the National Health Insurance Scheme (NHIS) under the National Health Insurance Act 650 (revised to Act 852 in 2012). The activities of the NHIS are supervised by the National Health Insurance Authority (NHIA). The implementation of the NHIS is a major step towards universal health coverage for all Ghanaians as the NHIS grants exemptions for various categories of individuals to promote their use of health services. These categories include: pregnant women, children under 18 years of age, elderly people 70 years and above, pensioners under the Social Security and National Insurance Trust, the indigent (poor and vulnerable) who must meet a certain criteria, and recently, those with mental health disorders. Pregnant women, indigents and persons with mental health disorders are not required to make any payment as processing fees before being registered into the NHIS; however the other exempt groups must pay a processing fee.

### The free maternal health policy under the NHIS

In 2008, the exemption for pregnant women was repackaged and branded as the ‘free maternal health policy’. The policy sought to enhance the utilisation of ANC, skilled attendance at childbirth and postnatal care. Under the policy, following confirmation of pregnancy by a medical officer or midwife/nurse and registration into the NHIS, a pregnant woman is immediately entitled to free health services for the pregnancy, during labour and birth and up to three months postpartum.

Empirical evidence showed a strong positive relationship between the provision of fee exemptions or health insurance coverage and the use of ANC and skilled attendance at childbirth leading to positive health outcomes [20, 21]. In Malawi, for instance, fee exemption in mission health facilities brought about a 15% increase in the mean proportion of participants who had at least one ANC visit during pregnancy [22]. On the other hand, studies have established that households still incur costs despite fee exemptions. For example, a study in Bangladesh showed that the majority of women incurred direct OOP payments for registration, consultation, laboratory test, drugs, transportation, and other related expenses in the course of seeking maternal health services [23]. Similarly, some households in India were found to have made significant OOP payments for maternal health services, even though these services were meant to be free [24]. A previous study in the Kassena-Nankana municipality, Ghana, where this study was undertaken, revealed that cost of transport alone accounted for about 32% of the total expenditure incurred by families for the treatment of maternal complications during childbirth [25]. Thus, direct OOP payments in whichever form could still be a serious barrier to the use of health services [16–18].

It is unclear whether the free maternal health policy in Ghana altered OOP payments during pregnancy. Internationally, several quantitative studies have examined costs for pregnancy and childbirth combined. For example, a quantitative study in Ghana has previously estimated the cost of maternal complications during childbirth in Northern Ghana [25]. Our mixed methods study was focused on care during pregnancy. The aim was to explore views on costs and actual payments made during pregnancy under the free maternal health policy. In addition, the source of funding for the payments was also explored. Views of women, midwives and nurses, as well as health insurance managers/directors were studied.

## Methods

### Study design

A convergent parallel mixed methods design was used, involving quantitative and qualitative data collection approaches [26]. In a convergent parallel mixed methods design, data collection and analysis for the quantitative and qualitative studies are carried out in parallel, that is, at the same time. The findings are then integrated to ensure a comprehensive analysis of the research question [26]. The quantitative component used a structured questionnaire among women who had given birth in health facilities and at home. The qualitative component involved focus group discussions (FGDs) and in-depth interviews (IDIs), using semi-structured interview guides with similar groups of women. The FGDs were held with women who gave birth in health facilities and at home after the use of maternal health services. The IDIs were held with midwives and nurses, and health insurance managers/directors. The FGDs and IDIs were considered appropriate for capturing the views of women, midwives and nurses, and managers/directors on the operations of the free maternal health policy. Data collection was carried out over a period of six months (March-August, 2016).

The study received ethics approval from the Ethical Review Board of the Navrongo Health Research Centre in Ghana (NHRCIRB217) and the Human Research Ethics Committee of the University of Technology Sydney, Australia (ETH16-0263). All participants provided consent to participate. Written permission was also obtained from the district and regional directors of health services representing the Kassena-Nankana municipality and the Upper East regional health directorate, as well as from the management of the health facilities where the study was conducted.

### Study area

The study was conducted in the Kassena-Nankana municipality of the Upper East region of Northern Ghana. The study area was selected as it is considered one of the poorest in Ghana. In 2010, around one fifth of the population of 108,000 were declared as poor with agricultural activities (65.4%) dominating the economy [27].

The municipality has one main hospital and two health centres. In addition, there are 17 community-based health planning services (CHPS) compounds, one private clinic and one health post (mission-based). The CHPS compounds are relatively small health facilities located in deprived and remote communities. The CHPS compounds provide basic health services to members of the communities in which they are located [28]. Apart from the resident nurse, some of the CHPS compounds are staffed with midwives to provide antenatal, childbirth and postnatal services. A few of the CHPS compounds in the municipality are provided with basic laboratories, delivery rooms, pharmacies, electricity and water. The majority are without these facilities. At the time of the study, none of the CHPS compounds had an ultrasound equipment. The midwives and nurses stationed in the CHPS compounds without the above-mentioned facilities do their best to provide whatever services are available and then refer pregnant women to the main hospital and other private laboratories and pharmacies for other required services. Some health centres also provide maternal health services.

### Sample size determination for the survey

The study sought to ensure representativeness for women who gave birth in health facilities and at home. In this region, most women attended ANC services although not all attended a health facility to give birth. Therefore both groups of women were needed to provide a representative sample. For the survey, the formula proposed by Gorstein et al. was used to calculate the sample size for a proportion in a single cross-sectional survey [29]. The formula is shown below, where 1.96 = 95% confidence interval, p = expected proportion, DEFF = estimated design effect and d = desired level of absolute precision:

$$n=\frac{1.96^{2}p(1-p)(DEFF)}{d^{2}}$$

The proportion of skilled attendance at childbirth in Ghana was estimated to be 60.5% [30] and this was used to determine the sample size for women who gave birth in health facilities. Given a 95% confidence interval with a width of ±10 (level of absolute precision), design effect of 2, 60% proportion of skilled attendance at childbirth, and a non-response rate of 5%, the calculated sample size for women who gave birth in health facilities was found to be 194. The same parameter was assumed for determining the sample size for women who gave birth at home, but with a proportion of 40% instead, leading to a sample size of 194. Thus two groups of women were recruited for the survey; women who gave birth in health facilities and women who gave birth at home after utilising ANC and other health services. Overall, 388 women were required, but the study recruited a total of 406 women to accommodate withdrawals.

### Participants for the qualitative component

Ten (10) FGDs were carried out with women who gave birth in health facilities (n = 7) and at home (n = 3). Each discussion group comprised of about 5–12 members. Twenty-eight IDIs were conducted, constituting 25 IDIs with midwives and nurses and one IDI each with the district manager of the NHIS, the regional director of the NHIA and a deputy director at the headquarters of the NHIA. The selection of the participants for the IDIs was purposive, interviewing frontline health providers as well as leaders of the NHIS/NHIA.

### Recruitment of study participants

All health facilities in the study area providing antenatal, childbirth and postnatal services were visited daily to identify women who had recently given birth. Women were recruited only after they had been discharged home from the health facilities. A list with contact details for women who gave birth at home was generated from the database of the Navrongo Health Surveillance System located at the Navrongo Health Research Centre, Ghana. That list was used to trace and recruit the women who gave birth at home. In addition, the study used the records for postnatal attendance to trace women who gave birth at home. Because the survey recruited women who had already given birth at home, 1^st^ January, 2016 was set as the starting date for inclusion in the survey. Women who gave birth earlier were ineligible to participate in the survey. This was done to help minimise recall bias. It took about 30–45 minutes to complete each questionnaire of the survey. Research assistants administered the survey and were supervised by the main investigator.

With the assistance of midwives and nurses at the health facilities, women who gave birth and had come for postnatal services were invited to participate in the FGDs. The lists kept by the health facilities of women who gave birth at home as well that generated from the database of the Navrongo Health Surveillance System were used for inviting women to participate in the FGDs. The FGDs were conducted at the premises of health facilities, without health providers. This enabled privacy and free expression of views.

The midwives and nurses who were directly involved in the provision of maternal health services in the various health facilities were identified and invited to take part in an in-depth interview. The interviews with the midwives and nurses were held in secured rooms in the health facilities in which they worked. The health insurance manager responsible for the Kassena-Nankana municipality, the director for the NHIA Upper East regional office and a deputy director at the headquarters of the NHIA also participated in the IDIs. The IDIs were held in the offices of the managers/directors. All the IDIs were conducted one-on-one basis. Each FGD or IDI lasted 45–120 minutes. The main investigator conducted all the FGDs and IDIs.

### Study variables

Questions for the study covered topics ranging from socio-demographic characteristics of participants to challenges of the health system that affected the use and provision of maternal health services. Table 2 outlines the topics covered and the particular study components.

**Table 2:** Topics covered for the study.

Topics covered	Study component
Socio-demographic characteristics	Survey with women
Type of health facility visited during pregnancy	Survey and FGDs with women
Health and related services received at the facility	Survey and FGDs with women
Direct OOP payments for outpatient health services	Survey and FGDs with women, IDIs with midwives/nurses and health insurance managers
Direct OOP payments for food and transportation	Survey and FGDs with women, IDIs with midwives/nurses and health insurance managers
OOP Payments for hospitalization	Survey and FGDs with women, IDIs with midwives/nurses and health insurance managers
Source of financing payments	Survey and FGDs with women, IDIs with midwives/nurses and health insurance managers
Workings of the free maternal health policy	Survey and FGDs with women, IDIs with midwives/nurses and health insurance managers
Suggestions for improving health services	Survey and FGDs with women, IDIs with midwives/nurses and health insurance managers
Challenges of the health system	Survey and FGDs with women, IDIs with midwives/nurses and health insurance managers

OOP payments in this context refers to direct payments made by women or their partners/family members for the use of health services. The OOP payments for the survey were classified as direct medical and direct non-medical expenditure. Table 3 explains the cost components captured under direct medical and direct non-medical expenditure.

**Table 3:** Direct medical and direct-non medical expenses.

Outpatient	Hospitalisation (Inpatient)
**Direct medical expenses**	**Direct medical expenses**
Antenatal folder fee (ie. antenatal record)	Laboratory test
Consultation	Drugs
Laboratory test	Blood transfusion
Drugs	Bedding
Blood transfusion	
**Direct non-medical expenses**	
Feeding	
Transport	

Direct medical and direct non-medical costs were estimated by aggregating the costs from which means and standard deviations were determined. Hospitalisation was defined as those who were admitted in a health facility for longer than 12 hours and the costs were a summation of medical costs, laboratory costs, and bed costs paid by either the woman or partner/ family member during the admission. The recall period for the cost component covered the entire period of pregnancy (7–9 months). Indirect costs (for example; opportunity costs due to time spent at health facilities) were not explored for the survey as this was beyond the scope. Women were the main respondents in the survey but where a particular woman could not provide the needed information, especially on cost, her partner/family members could assist.

The study determined the impact of the OOP payments on the women by estimating the proportion of the payments from the average annual household income of the Upper East region, which was obtained from the Ghana Living Standards Survey Report of the sixth round [2]. Data was not available for the study area itself. We used 9/12th of the average annual household income to correspond to the duration of a normal pregnancy (7–9 months), with the assumption that all women attended ANC at the recommended time. A 9/12th average annual income for the region was estimated to be GH¢5,430.40 (US$2,755.40) for 2012/13. The study determined catastrophic OOP payments as well for the women. OOP payments are considered to be catastrophic if they are equal to or above a certain predetermined cut-off point or threshold of household resources in terms of income, expenditure or consumption. Catastrophic OOP payments affects the welfare of households as they have to forfeit the consumption of certain essential goods and services as a result of the payments. Studies have used different thresholds such as 5%, 10%, 15%, 20%, and 40% for the determination of catastrophic OOP payments [25, 31–33]. This study used 5% threshold of 9/12 average annual household income. If the total OOP payments (direct and direct non-medical expenses) exceeded that threshold (5%), it was considered to be catastrophic.

The costs data for the study was reported in Ghana cedis, but converted into US$, using an exchange rate of US$1 = GH¢1.9708 (2013 exchange rate) as existed in the Ghana Living Standards Survey Report of the sixth round.

### Study process for the qualitative component

The semi-structured interview guides for both the FGDs and the IDIs were developed in English. The questions for the FGDs were later translated into the two dialects (Kasem and Nankani) spoken in the study area. The guides for the IDIs were not translated because all the midwives and nurses, and managers/directors spoke and understood English. All questions for the FGDs and IDIs were piloted and changes were made accordingly. The discussions and interviews were audio-recorded with the permission of participants, with the exception of one interview by a health insurance manager who asked not to be recorded. Extensive field notes were taken for all the discussions and interviews alongside the recordings.

The FGDs and IDIs took a flexible approach. Emerging issues were probed further to ensure that pertinent issues were not left out. During the FGDs, the facilitator made an effort to ensure all participants, particularly those seen not to be active discussants, were able to present their views. At the end of the discussion or interview, issues were summarised and presented back to participants to ensure they were as expressed, and new issues that emerged were incorporated into the guides for the next discussion or interview. The discussions and interviews ended when there was saturation after prompts from the investigator.

### Data analysis and management

Survey data were collected electronically using SurveyCTO Collect v2.10 application. Data analysis was carried out using STATA 14. Data were cleaned by checking frequencies to identify outliners as well as missing data. Descriptive statistics were used to describe the background characteristics of the participants and other variables.

The audio recordings for the qualitative study were transcribed or translated verbatim into English. The transcripts and field notes were read several times to immerse the researcher in the data. For validity and accuracy purposes, the main investigator listened to a number of recordings, comparing that to the transcripts and any discrepancy was corrected before coding. All transcripts and interview notes were read and reviewed further with hand written notes on each transcript highlighting key issues. A coding structure was then developed and applied to identify themes and sub-themes which were presented using tables. For assurance that the themes reflected the data, the particular data section(s) for each theme was re-examined with alterations made when necessary. The findings therefore reflected the themes and included essential key quotes from the participants (S1 and S2 Files).

## Results

### Socio-demographic characteristics

A total of 406 women participated in the survey. The mean age for the women was 27 years, majority (66.7%) were under 29 years. Most were married (95.1%), more than 70% had only basic education (that is, up to completion of junior high school) or no formal education, and 38.2% were involved in farming. About one third of the participants (31.5%) were first time mothers (Table 4).

**Table 4:** Socio-demographic characteristics of participants.

Variable	Overall total	Number	Percent (%)
		406	100
**Age**	<20	41	10.0
	20–24	103	25.4
	25–29	127	31.3
	30–39	120	29.6
	40+	15	3.7
**Marital status**	Single	20	4.9
	Married	386	95.1
**Highest educational level**	No formal education	64	15.8
	Basic education (up to junior high school)	226	55.7
	Senior high/Technical education	67	16.5
	Tertiary	49	12.0
**Occupation**	Unemployed	29	7.1
	Trader	79	19.5
	Farmer	155	38.2
	Public/Civil servant	46	11.3
	Student	42	10.3
	Other	55	13.6
**Religious background**	Traditional	20	4.9
	Catholic	164	40.4
	Protestant	192	47.3
	Muslim	30	7.4
**Ethnicity**	Kasem	239	58.9
	Nankam	135	33.2
	Other	32	7.9
**Number of births**	1	128	31.5
	2	103	25.4
	3	79	19.5
	4 or more	96	23.6

### Type of health facility visited during pregnancy

**Fig 1:**
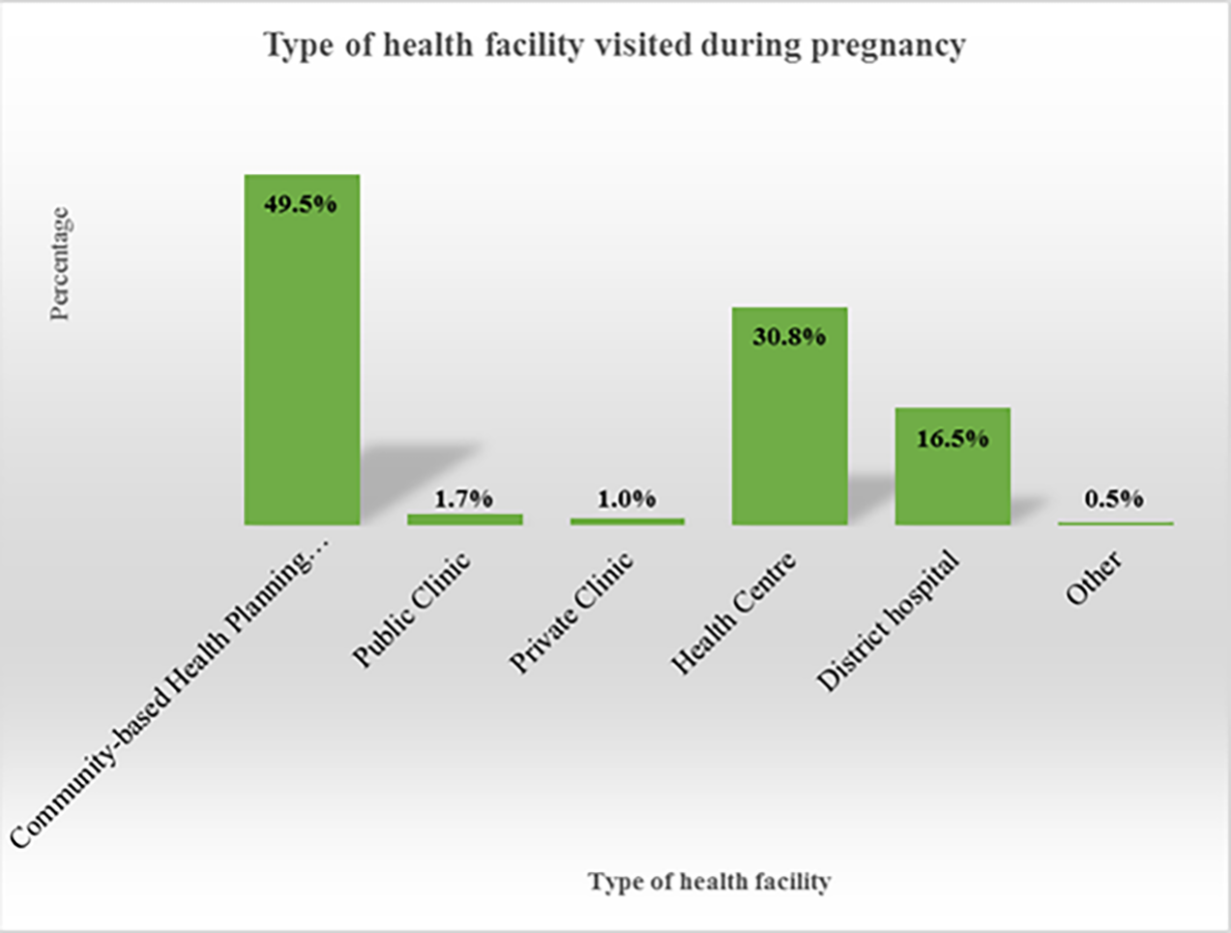
Type of health facility visited during pregnancy.

Almost half of the participants (49.5%) utilised services from the CHPS compounds (Fig 1 & S3 File). About a third of the women also used services from health centres (30.8%).

From the FGDs, most of the participants indicated that they were utilising the CHPS compounds for ANC and other services during pregnancy. A participant said;

*“We always go to the …… clinic (referring to CHPS compound) for ANC and other health issues”* (FGD, woman).

### Health and related services received during pregnancy

Virtually all women (100%) had undergone physical examination (including weight, blood pressure, and heart rate). Also, 95.0% and 98.5% of the women respectively, had received obstetrical examinations and ultrasound scans (Table 5).

**Table 5:** Health and related services received during pregnancy.

Services received during pregnancy		Number	Percent (%)
Physical examination (including weight, blood pressure, heart rate)	Yes	406	100
Obstetrical examination (abdominal palpation and vaginal examination)	Yes	386	95.0
Ultrasound scan	Yes	400	98.5
HIV/STD testing	Yes	377	92.9
	Don’t know	6	1.5
Blood test	Yes	397	97.8
Nutritional supplements	Yes	392	96.5
Tetanus vaccine	Yes	396	97.5
	Don’t know	10	2.5

### Coverage of expenses by the free maternal health policy during pregnancy

From the quantitative aspect, 90% of the women felt that the free maternal health policy under the NHIS did not cover all expenses. Findings from the FGDs and the IDIs with the midwives and nurses also supported this opinion. A participant in the FGD said;

*“We pay for other services*, *except for ANC and delivery services that the health insurance covers”* (FGD, woman).

A midwife also said;

*“These women make payments for some of the health services*, *especially for drugs and laboratory services*. *And then buying rubber*, *pads; some of those small*, *small things that they will have to use during the delivery*, *they are not covered”* (IDI, midwife).

However, the managers/directors of the NHIS/NHIA felt that the free maternal health policy under the NHIS did cover all expenses for maternal health services. A manager stated that;

*“All health services and approved drugs are provided for free for pregnant women in NHIS accredited health facilities”* (IDI, manager).

The managers/directors reported that only the cost of transportation to and from health facilities were not covered under the maternal health policy. One of the directors said;

*“No*, *transport is not covered*. *I am told if even when an ambulance takes a patient from one place to the other*, *the patient incurs that cost”* (IDI, director).

The midwives and nurses concurred that the cost of transportation was borne by the women and their relatives. A nurse indicated;

*“Transportation like this I’m aware is not covered*. *Like if you are going to refer somebody*, *it’s not covered–they (women) will have to pay”* (IDI, nurse).

### Actual OOP payments incurred during pregnancy

The majority of the women made direct OOP payments for laboratory tests and drugs representing 87.4% and 50.0% respectively (Table 6). The mean for total direct medical expenses was GH¢18.0 (US$9) and GH¢16.50 (US$8.40) for direct non-medical expenses. The combined mean for direct medical and direct non-medical expenses was GH¢17.50 (US$8.90) for outpatient attendance. The mean OOP expenditure for hospitalisation was GH¢24.50 (US$12.40). Overall, the mean OOP expenditure incurred during pregnancy was GH¢17.50 (US$8.90), reflecting as 0.32% of 9/12th average annual household income of GH¢5,430.40 (US$2,755.40) for the Upper East region in 2012/13. Approximately 2% (n = 9) of the women had used more than 5% (threshold) of 9/12th average annual household income and thus faced catastrophic OOP payments.

**Table 6:** OOP expenditure incurred during pregnancy.

OOP expenditure incurred during pregnancy			Mean	Std dev.
	Number	% of women	GH¢ (US$)	GH¢ (US$)
(1) Direct medical expenses (outpatient)				
-Folder fee	5	1.2	11.60 (5.90)	11.30 (5.70)
-Consultation	1	0.2	20 (10.10)	-
-Laboratory test	355	87.4	38.60 (19.60)	46.80 (23.70)
-Drugs	203	50.0	35.70 (18.1)	67.70 (34.40)
-Blood transfusion	7	1.7	64.30 (32.60)	36.90 (18.70)
**Total direct medical expenses**			**18 (9.10)**	**22.80 (11.60)**
(2) Direct non-medical expenses				
-Feeding	50	12.3	15.30 (7.80)	11.50 (5.80)
-Transport	44	10.8	18.70 (9.50)	30.50 (15.50)
**Total direct non-medical expenses**			**16.50 (8.40)**	**16 (8.10)**
Total direct and direct non-medical expenses (1+2)			**17.50 (8.90)**	**18.60 (9.40)**
(3) Hospitalisation expenses (inpatient)	8	2.0	**24.50 (12.40)**	**32 (16.20)**
**Total direct expenses (1+2+3)**			**17.50 (8.90)**	**18.50 (9.40)**

Exchange rate of GH¢1.9708 = US$1

The women attended the health facilities with the understanding that all the expenses would be covered by the NHIS. For example, one woman said;

*“On my first attendance*, *I didn’t know I had to pay for the testing*, *so when I got there I was asked to pay GH¢15 (US$7*.*60) and I went home*, *the next day I went back and they said the same thing*, *it was on the third day that I was able to raise the money to pay”* (FGD, Woman).

The midwives and nurses recognised that women visited the health facilities with the expectation that they would not be paying for services and drugs. Explaining this, a midwife said;

*“……*.*because she (the pregnant woman) thinks that she is under health insurance*, *she is covered under everything*, *she comes here and you tell her to buy something*, *she is not prepared for that……”* (IDI, midwife).

### OOP payments for anti-malarial drugs and ultrasound scan

In the FGDs, women explained that they were required to buy anti-malarial drugs as well as pay for ultrasound scan. A quote from one of the participants was;

*“There were some drugs that they say they are for malaria*, *first it was for free but now when you attend the facility*, *they will ask you to go and buy at GH¢1*.*50 (US$0*.*80)”* (FGD, woman).

The midwives and nurses also recognised that women were often required to buy anti- malarial drugs as well as pay for ultrasound scan services. A midwife reported;

*“And the other thing too is the SP drug (Pyrimethamine-sulfadoxine) which we were giving free; now we have to ask some of them to go and buy because it’s currently not available*, *but beneficial*..... *We also request the women to go outside for scanning services and other tests*, *which they’ve to pay for”* (IDI, midwife).

Some of the women could not afford payment for the drugs and ultrasound scans. For example, a midwife said;

*“I can remember I asked one client to go and buy the SP drug*, *she didn’t tell me whether she had money or she didn’t have money*. *She just quickly went back home*, *remained there; for 2 months she didn’t come back for weighing*. *So later on I traced up only for her to tell me that*, *the drug I asked her to buy she couldn’t afford it*. *So she’s waiting*, *when she’s able to buy the drug*, *then she will come for weighing again……*.*”* (IDI, midwife).

In addition, because of the need to make direct OOP payments, some of the women did not undergo an ultrasound scan despite this being recommended. A midwife made the following statement;

*“I have a client who has never taken the scan…until delivery*, *upon referrals–she’ll never go”* (IDI, midwife).

The midwives and nurses understood that poverty meant some women could not afford the health services required. A midwife explained;

*“……the poverty level is very high*, *we all know that*. *Some we have pity for them–when you see them and follow them up to their houses*, *you’ll see what is happening under the ground*. *It’s not their making*. *Even the flour water for them to take is a problem”* (IDI, midwife).

Some women felt that the payments they had made in terms of drugs and other services was more than what the NHIS had paid for on their behalf for use of maternal health services. A participant said;

*“What we go to buy is more than what the NHIS offers us”* (FGD, woman).

Due to the payments that the women were still making for the use of maternal health services, midwives and nurses questioned the effectiveness of the free maternal health policy in the elimination of OOP payments. A midwife reported;

*“They (policy makers) have come out that free maternal health…but how free is it*?*”* (IDI, midwife).

A midwife explained further;

*“…… there are certain “peti-peti” things that we still ask the women to buy*, *like the SP drugs …under the free maternal health policy”* (IDI, nurse).

Notwithstanding, most of the women were positive about the benefits of the free maternal health policy. A participant in the FGDs said;

*“They used to give flour*, *I will not lie*, *and I received it once*. *It was flour and oil but I received the flour and bed nets and also the routine drugs*. *These are all free and we are happy with that”* (FGD, woman).

The flour and oil are usually given to supplement the nutritional needs of women and their babies. These food products are donated by the World Food Programme and the United Nations Children’s Fund.

Despite all the concerns, the managers/directors were optimistic about the benefits of the free maternal health policy to health service delivery. A director said;

*“The free maternal health policy is one of the major policies under the NHIS which has helped improve maternal health care and reduced infant deaths or child mortality*. *Health providers have also benefitted a lot from our reimbursement*, *the only challenge is the late reimbursement of health facilities*. *For this we are helpless”* (IDI, director).

### Source of financing health expenses

**Fig 2:**
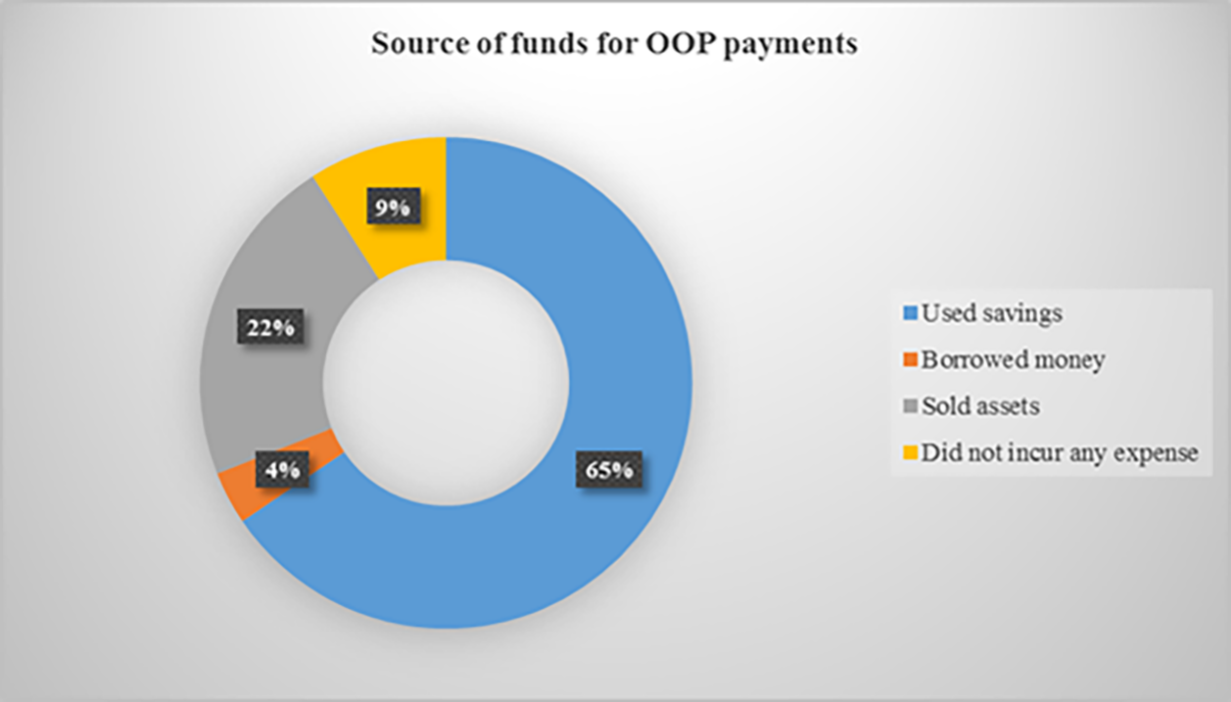
Source of funds for OOP payments.

Women used various sources of funding to meet the expenses incurred during pregnancy (Fig 2 & S3 File). In the survey, over two-thirds (65%) of the women said they had to use their savings to finance the expenditure, whilst 22% indicated that they sold assets to meet the expenditure.

Women also explained how their families sold assets in order to finance the payments for drugs and ultrasound scans. These assets include domestic animals like sheep, goats, guinea fowls and fowls, and foodstuffs. A participant said;

*“My husband took an animal to the market to sell so that I can get money to pay for the scan”* (FGD, woman).

### Suggestions for improving maternal health services during pregnancy

**Fig 3:**
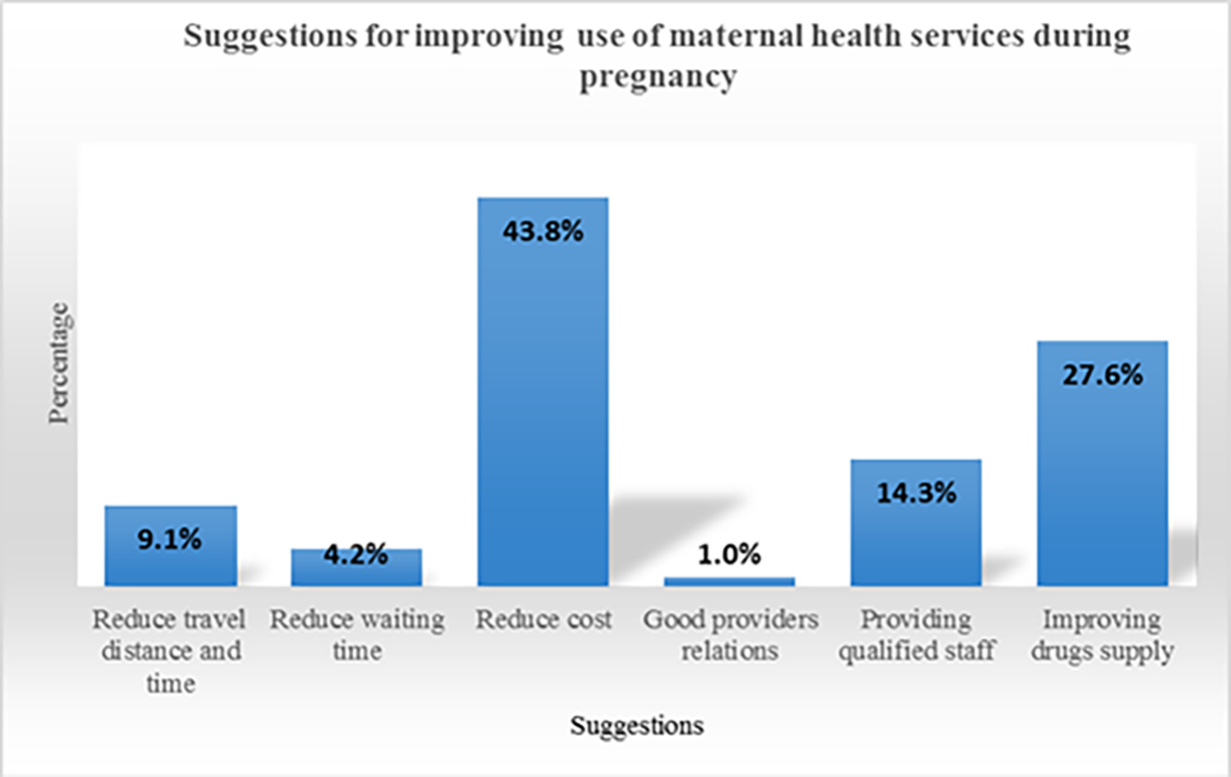
Suggestions for improving use of maternal health services during pregnancy.

The survey asked women for suggestions on how to improve access to maternal health services under the free maternal health policy. The lead response was the reduction of cost (43.8%), followed by improvement in drugs supplies (27.6%) (Fig 3 & S3 File). Providers’ relations, travel distance and waiting times were not serious issues hindering the use of maternal health services.

The women in the FGDs said efforts should be directed at reducing OOP payments for use of maternal health services. For example;

*“The payment for the scanning and drugs should be looked at…”* (FGD, woman).

In the IDIs, midwives and nurses expressed concerns about the inability of the NHIS to cover the cost of transportation to and from health facilities. They felt that the NHIS’s benefit package should include the cost for referral of emergency cases to higher health facilities. A nurse said;

*“And if the health insurance can also help so that when we are referring them (women) like this*, *the ambulance*, *it should be free–they should not be requesting money from them–It would have also helped”* (IDI, nurse).

## Discussion

The study showed that the women, midwives and nurses felt the free maternal health policy did not cover all expenses associated with the use of maternal health services. Proportionally, 87.4% and 50.0% of the women made direct OOP payments for laboratory tests and drugs respectively. A mean OOP cost of GH¢17.50 (US$8.90) was incurred for use of maternal health services during pregnancy. The FGDs and IDIs indicated that anti-malarial drugs and ultrasound scan services were paid by OOP too, but some of them were unable to afford paying due to poverty. The women who made the payments used savings as well as sold assets. In contrast, the managers/directors of the NHIS/NHIA argued that the NHIS did cover all basic health expenses, except the cost of transport. Both women and insurance managers/directors however, acknowledged the benefits of the policy.

The mean OOP payments for use of maternal health services during pregnancy was GH¢17.50 (US$8.90) and estimated as 0.32% of 9/12th average annual household income of the region. Besides 2% of the women faced catastrophic OOP payments which could affect their consumption of basic goods and services in the midst of the free maternal health policy. The low proportion of OOP payments share of the average annual household income as well as the low proportion of the women who faced catastrophic OOP payments might be explained by the fact that some of the women were unable to afford payment for some services. Most individuals in the study area are not formally employed, instead they are engaged in peasant agricultural activities (65.4%) with limited cash income, with one fifth of the population of 110,000 declared as poor [27]. Thus OOP payments of any magnitude might be unaffordable and to some extent, adversely affect the economic fortunes of some of the households.

The NHIS’s benefit package covers 95% of disease conditions in Ghana, whilst the free maternal health policy purports to allow for the free utilisation of maternal health services including drugs, ANC, childbirth (normal and assisted), caesarean section and postnatal care [34]. However, the finding on OOP payments were a consequence of the unavailability of drugs and other essential supplies in the health facilities, as found elsewhere [24, 35]. The NHIS is required to make timely payments for claims submitted by health facilities for use of health services by registered clients. Unfortunately, at the time of the study, health facilities were not reimbursed of the last seven months and this had affected their ability to procure drugs especially. The phenomenon generated stock-outs of drugs in the health facilities, explaining why health providers had to write out prescription forms for women to buy drugs outside health facilities.

Another systemic challenge was the lack of well-equipped laboratories and qualified personnel, particularly in the lower level health facilities [3, 36] in the study area. The main hospital is the only facility with a standard laboratory. Even for the district hospital, due to lack of funds resulting from the delay in reimbursement by the NHIS, the facility is unable to procure the necessary test kits and reagents for use. For these reasons, women were consistently referred to private laboratories for tests and scans, triggering additional OOP payments.

In the study area, a cross-sectional study on cost of maternal complications during pregnancy revealed that households spent a median of US$32.03 for the treatment of maternal complications [25]. Two reasons might account for the difference in the mean OOP expenditures between that study and ours. Generally, the cost of treatment for maternal complications is usually higher than that for pregnancies without complication. Secondly, the previous study added productivity costs, which was not captured for this study. Nationally, our finding is consistent with a recent report released by the Ghana Statistical Service on the purchase of drugs outside health facilities under the NHIS. The report highlighted average OOP payments for drugs outside health facilities to be GH¢34.32 (US$ 17.40) versus GH¢33.84 (US$17.10) for drugs obtained from health facilities [2]. It is important to note that the reported averages represent the use of health services by the general population including pregnant women.

Similarly, a study in a rural setting in Nigeria, West Africa, showed that women and their families spent between US$9 and US$99 for use of maternal health services [37]. In India too, a recent study reported that households incurred a mean OOP expenditure of US$ 26 in public health facilities for the utilisation of maternal health services [38]. These studies bear resemblance to our study, given that the mean OOP costs were for use of maternal health services in public health facilities in the midst of fee exemptions or health insurance.

The finding on payments made by the women violates the core principle for implementing the free maternal health policy. As a result of the OOP payments, some women were unable to buy anti-malarial drugs as well as undergo ultrasound scans in particular. This may have serious implications for the health of the women involved and their unborn babies. The women’s inability to utilise maternal health services as a result of OOP payments would setback the agenda of reducing maternal mortality in the country. It would also stall the process for the achievement of universal health coverage as envisioned in the sustainable development goals [39]. The NHIS should endeavour to make early payments for claims submitted by health facilities. It is also suggested for the NHIS to include the cost of transportation for emergency cases in its benefit package to assist reduce the financial burden of women who would require referral. On the part of government and donors, we recommend an increased funding to the health sector, especially support for infrastructural and logistical improvement in the health facilities.

If these suggestions are acted upon, payments for maternal health services during pregnancy might be reduced or eradicated altogether. This will enable all women to be able to use maternal health services when required, leading to improved health outcomes in terms of reduced maternal mortality. It will also provide universal health coverage for all women at the long run.

## Study limitations

The combination of quantitative and qualitative methods ensured an in-depth analysis for the achievement of the research goal [26]. The findings from each component of the study assisted shed light, especially the qualitative study helped illustrate the findings of the quantitative through the use of key quotes. However, the following should be noted. The women were interviewed after the use of ANC and other health services, thus there is the chance of their inability to remember all details of their health seeking experience during pregnancy. Even though the quantitative component was conducted within eight months after giving birth, recall bias cannot be eliminated completely. The study did not examine the full costs related to pregnancy, since the loss of productivity was not determined and included. So the mean OOP payments might be an underestimations of the actual cost incurred during pregnancy.

## Conclusion

The study showed women made OOP payments under the free maternal health policy. Women paid for drugs, especially anti-malarial drugs and ultrasound scan services out of health facilities during pregnancy, potentially leading to inadequate care and health implications for women and their unborn babies. The NHIS should make timely payments for claims submitted by the health facilities, to assist reduce or eliminate the payments associated with the utilisation of maternal health services. The NHIS should also support women on emergency referral to higher health institutions to help reduce their financial burden. In addition, government and donors should increase funding for infrastructural and logistical improvement in health facilities. These measures when adopted will make all women be able to use maternal health services when needed, thus improving health outcomes including the reduction of maternal deaths. It could also enhance the move towards universal health coverage.

## Supporting information

S1 File Excerpts for affordability_Midwives & Nurses.docx: Key quotes from midwives and nurses. (DOCX)

S2 File Excerpts for affordability_Women.docx: Key quotes from women. (DOCX)

S3 File Supporting file for figures.xlsx: Conversion for the figures. (XLSX)
