# Supplementary material for: Has the free maternal health policy eliminated out of pocket payments for maternal health services? Views of women, health providers and insurance managers in Northern Ghana
Source: PLoS One. 2018 Feb 1;13(2):e0184830. doi: 10.1371/journal.pone.0184830 (PMC5794072; doi:10.1371/journal.pone.0184830)
Supplement: S1 File — (DOCX) [file pone.0184830.s001.docx]

**Key excerpts for affordability of maternal health services- Midwives/nurses**

1. **Scanning not covered by the NHIS**

*“And with the scan, it’s not being covered under the free maternal health policy? The facilities don’t have them. If they do have they would have done…they will do a scan, they take a…at GHC 28…or if the need arise we’ll…we don’t have those things…I have a client who has never taken the…until delivery, upon referrals – she’ll never go”.*

*“For the challenges there are a lot sometimes the drugs; for a very long time now we’ve not…in our sub-district, so I write for them to go and buy; and where is the free maternal health service?”.*

*“For example this scan you ask them to take scan, the first time and then 32 and 36 and some of them they don’t have money and health insurance is not covered. One thing I forgot, scan is not covered by health insurance and some always don’t have money but you will really see that they need to take a scan on their pregnancy but you cannot do anything to help them so that one if she doesn’t take the scan where is the quality you are providing because that one you don’t know how she is faring but you are agreeing to manage her like that because she doesn’t have money and health insurance is not covered so you also go by that”.*

1. **Inability to afford ultrasound scanning**

*“I have a client who has never taken the scan…until delivery, upon referrals – she’ll never go”*

*“…………as I said the ultrasound is also to be done free under the health insurance policy but ever since I have started working here since 2010 it is not free, the clients go outside to access that service and they bring it to us”.*

*There are some clients they will never do an ultrasound till they deliver. That is my problem. When you go down south they pay but the three northern regions because we are tagged as poor they say they don’t have money”.*

*“…I will say that the scan is not covered. The scan even though is covered the hospital doesn’t have the scanning machine.”*

*“When I was in the labor ward we use to do the scanning ourselves but since I came to the ANC the women go outside for it”.*

*“……….most of the women they are always complaining that they are always going outside to do the scan. For now that what I can say and there are some times some of the things are in short supply, like the drugs and they will have to go and buy and they will be complaining these are the things that make our work difficult but when every is there the work goes smoothly.*

*no please. For this month what the women do outside our facility is the scan and one drug that they buy apart from that most of the things we only write in the folder that client has been taken care of and then fill the health insurance and they go for it”.*

*“And again if you ask them to do the scanning some of them are always complaining that they don’t have money”.*

*“We also request the women to go outside for scanning services and other tests, which they’ve to pay for”*

1. **Poverty and the use of health services**

*“The challenges are “go and do your lab examination”, your own transport, send her to hospital at the time she’s helpless, at the time she’s in pain. But these are things we talk to them before they even get due but still they come. They come because she doesn’t have, and she’s looking unto God to work – so whatever will happen will happen”.*

*“Yes. The poverty is high. When they come, bring transport, we’ll be carrying the wife on a motorbike then you support at the back then we get there. Some don’t even have a bicycle.”*

*“……the poverty level is very high, we all know that. Some we have pity for them –* *when you see them and follow them up to their houses, you’ll see what is happening under the ground. It’s not their* *making. Even the flour water for them to take is a problem”*

*“And our people here are very poor. Even the basic basic things, even the common rags that they even bring in to deliver, some cannot even bring those things. It’s a very big challenge. Some even after delivery, to get food to eat is a problem”.*

1. **Transportation and other things are not covered by the NHIS**

*“Transportation like this I’m aware is not covered. Like if you are going to refer somebody, it’s not covered – they will have to pay. They will have to pay for their own transport”.*

1. **What makes women not adhere to treatment regimen?**

*“…..we have a drug we call SP for malaria they are supposed to take it in front of you, you will observe but they don’t have…there is no water provided for them to always use and take so some of them you ask them to go and bring the water and take the drugs and they will go and not comeback”.*

*“Maybe she doesn’t have money for the water. Some will come they tell you they don’t have money with them some too they won’t tell you they don’t have money but they will go and wont comeback maybe she doesn’t have money but she didn’t tell you and come later maybe her next visit and she would have defaulted from that time. The time she gets money for the water and is coming maybe she will be coming for the third visit instead of the second visit so you have to take it like that. So if there is water at the facility that we give to the women to take the drug”.*

1. **Health insurance and payments for drugs and tests**

*“…….because she (the pregnant woman) thinks that she is under health insurance, she* *is covered under everything, she comes here and you tell her to buy something, she is not prepared for that……”*

*“These women make payments for some of the health services, especially for drugs and laboratory services. And then buying rubber, pads; some of those small, small things*

*that they will have to use during the delivery, they are not covered”*

*“But sometimes some of them they don’t even have money so asking them to buy I don’t think she will be happy because she thinks that she is under health insurance, she is covered under everything, she comes here and you tell her to buy something, she is not prepared for that even though we educate them on saving money for peti-peti things so if the drugs are available and she comes and she has them she will be happy and will come for our service always.*

*Something like scan, I know the free maternal health doesn’t cover that one so when the women are to do scan they go outside and do it”.*

*“The woman who just left here, she went and came back that because of the cost she could not do the test. So I was encouraging her to try and get some money, go and do it and come because it’s very important for her”.*

*“And I think we have some few challenges about this laboratory services, especially when they come for the first time for ANC services, there’re some challenges in it, because (1) you ask them to go, we don’t do the hemoglobin here, and we cannot even do the other…the stool and the urine; you have to refer them to the lab, either the hospital or central clinic, and when they get there too, they come back to tell us that they’re asked to pay a fee. Those who cannot afford the fee come. So in that sense, you’ll be forced to…you cannot also force a woman that it’s force, go and do it. So you’re treating the woman not knowing most of her health status like the sickling, if it’s not done, you don’t know whether if she’s a sicklier or not, you don’t know; her HB level, you cannot just look at her face and estimate; whether she’s having protein in urine, you cannot know, anything”.*

1. **Affordability of SP**

*“And the other thing too is the SP which we were given; now we have to ask some of them to go and buy because it’s beneficial to the woman”.*

*“I can remember I asked one client to go and buy the SP drug, she didn’t tell me* *whether she had money or she didn’t have money. She just quickly went back home, remained there; for 2 months she didn’t come back for weighing. So later on I traced up only for her to tell me that, the drug I asked her to buy she couldn’t afford it. So she’s waiting, when she’s able to buy the drug, then she will come for weighing again…….”*

*“For instance, the SP like this is an example – when they come we write for them to buy”.*

*“And the other thing too is the SP drug (Pyrimethamine-sulfadoxine) which we were giving free; now we have to ask some of them to go and buy because it’s currently no* *available, but beneficial…..”*

1. **Avoiding blame by nurses and midwives**

*“………….because of that anytime we want them to do something we ask “Do you have money to do this?”. When the fellow says no, we only write on the ANC card that you wanted the fellow to do this but the fellow said she cannot provide…if later on there is a problem they wouldn’t blame me that…because you tried”.*

1. **Free maternal health, but how free is it?**

*“The policy makers will have to review all things”. “They (policy makers) have come out that free maternal health…but how free is it?”*

*“But they are saying zero maternal deaths but there are certain things that are very difficult for us. If you are not very…at times you even have to put your hands inside your own pocket to let some of these issues resolve, if not you’ll pick up dead bodies which will not be fine”.*

*“…… there are certain “peti-peti” things that we still ask the women to buy, like* *the SP drugs …under the free maternal health policy”*

1. **Payment for transport by pregnant women**

*“Assuming we’re referring – we can’t manage and we’re referring, and it’s that, the Taxi is coming, they have to pay”.*

1. **Suggestion for improving use of maternal health services**

*“,…….. like scan like this when they come at least we were having it and it is working and they come it will be easy for them but at times when they come we refer them to private and some of the laboratory test they charge them. Some may come and they are to do the test but because they don’t have money they can’t do the test. So our lab too should be well equipped, you know if you go to a facility and they say this is not there go here, this is not there go there you won’t be happy. You want where you will go and everything will be available”.*

*“The other things are like, whilst they’re preaching free maternal health care, they should try and provide transport systems that can easily transport them”.*

*“And if the health insurance can also help so that when we are referring them (women) like this, the ambulance, it should be free – they should not be requesting money from* *them – It would have also helped”.*
