# Supplementary material for: Has the free maternal health policy eliminated out of pocket payments for maternal health services? Views of women, health providers and insurance managers in Northern Ghana
Source: PLoS One. 2018 Feb 1;13(2):e0184830. doi: 10.1371/journal.pone.0184830 (PMC5794072; doi:10.1371/journal.pone.0184830)
Supplement: S2 File — (DOCX) [file pone.0184830.s002.docx]

**Selected key excerpts from FGDs with women**

1. **Type of health facility visited**

*“We always go to the Gia clinic for ANC and other health issues”.*

## *“We have always been going for maternal health care service at the Korania health care center”.*

## *“Most of us attended this place (CHPS compound) for the ANC and for other services”.*

*“I started at Paga but later came here at Manyoro and I delivered here”.*

1. **All expenses covered?**

*“It is true when you come for ANC, it is always free but there are some smaller things that when you don’t have money, it is difficult”.*

*“I have not heard of it because you still spend money when you come to the facility you will still go and do scan which is not free, you will still go for lab and it is not also free”.*

*“We pay for other services, except for ANC and delivery services that the health* *insurance covers”.*

*“What we go to buy is more than what the NHIS offers us”*

1. **Payments incurred**

*“Scan we go and do it with our money but previously it was free. Now it is GH¢20 sometimes you don’t have money for it”.*

*“It is not free, when I went I spent GH¢20 on scan and the second time when I went it was GH¢30”.*

*“On my first attendance, I didn’t know I had to pay for the testing, so when I got there I was asked to pay GH¢15 (US$7.60) and I went home, the next day I went back and they said the same thing, it was on the third day that I was able to raise the money to pay”.*

*“There were some drugs that they say they are for malaria, first it was for free but now when you attend the facility, they will ask you to go and buy at GH¢1.50 (US$0.80)”.*

1. Source of financing

*“My husband took an animal to the market to sell so that I can get money to pay for the* *scan”.*

*“I used the money I saved for the payment of the drugs and the scan. Everything is finished now”.*

*“It was difficult, I had to use the money that I used for my petty trading to make the payment”.*

1. **Suggestions for improving maternal health services**

*“There are times that there will be just one nurse in the facility so if a woman is brought and another one too comes, the nurse will not be able to handle the two women so if they can give us more nurses so that they will attend to us quickly it will help. This way some will be able to go round the community and help us too”.*

*“The lab-test should also be looked at when I went there I thought it was free so when I went there the man there said it was 15 cedis so I had to go home and return on a market day to do the labs. The malaria drugs should also be free”.*

*“The payment for the scanning and drugs should be looked at…”.*

*“I will like to tell government that they should try and let the drugs reach us”.*

*“The payment for the scan should be looked at, when I was pregnant; I did not do the scan because I did not get money for the scan”*.

1. **Benefits of the NHIS**

*“They used to give flour, I will not lie, and I received it once. It was flour and oil but I received the flour and bed nets and also the routine drugs. These are all free and we are happy with that”*

*“The maternal health care policy is one of the major policies under the NHIS which was introduce to improve maternal health care and to reduce infant death or child mortality”.*
